# Fellow-Workers and the Roll of Honour

**Published:** 1915-03-13

**Authors:** 


### ROUND THE WORLD.

[The Editor invites Early Information and Contributions Marked "Fellow-Workers."]

### Fellow-Workers and the Roll of Honour.

THE Urgency Cases Hospital, which is being sent out with the approval of the French Government, should certainly accomplish a great deal towards saving life and limb in many instances where hitherto much time has been unavoidably lost in securing surgical aid. It is to be noted that the War Office has given leave to Major A. W. Mayo Robson, F.R.C.S., C.V.O., to go out with this hospital as consulting surgeon for a short time. Under his able direction will work Dr. J. A. Cairns Forsyth and Dr. K. E. Leverson Gower Gunn as surgeons; Dr. A. S. Robinson and Dr. W. H. Ogilvie as assistant surgeons; Dr. R. W. W. Vaughan as anæsthetist; and Mr. J. G. Everitt as radiographer and electrician.

MAJOR A. W. MAYO ROBSON, who was made a C.V.O. for his public services in 1911, is a distinguished representative of the school of surgery at Leeds, where for many years he served on the active staff of the General Infirmary (1884-1902). He was also professor of surgery in the Victoria University from 1890 to 1899, and has been Emeritus professor in the University of Leeds since 1904. An F.R.C.S. Eng., D.Sc. (Honours) Leeds, and a Knight of Grace of the Order of St. John of Jerusalem, Major Mayo Robson retired after an exceptionally successful career; now, as an officer of the R.A.M.C.(T.), his services are being found invaluable to our military forces.

MR. JOHN ANDREW CAIRNS FORSYTH is also a Leeds man and qualified M.B., Ch.B. in 1901; at the same time he holds the degree of M.Sc. Vict., and is an F.R.C.S. Eng. After qualification he held various important appointments in Leeds, including those of pathological curator and senior house surgeon to the General Infirmary, and assistant demonstrator in pathology and physiology in the University.

MR. WILLIAM HENEAGE OGILVIE is an Oxford and "Guy's" man, who two years ago qualified M.R.C.S., L.R.C.P., also taking the M.B., Ch.B. Oxon, after which he held the posts of assistant house surgeon and casualty officer at "Guy's." Mr. Ogilvie has had experience of fighting in the Balkans, and published some account of bullet wounds in Montenegro in the *Guy's Hospital Gazette* a year or two ago.

EXCELLENT work has been done by the 2nd Western General Hospital at Manchester, where Lieutenant-Colonel J. W. Smith and Major F. H. Westmacott, both of the Manchester Royal Infirmary staff, are respectively Commandant and Registrar-Secretary. According to an interesting report on the work of this institution in the *British Medical Journal*, over 730 operations have been performed there, whilst more than 1,000 patients have been submitted to *x*-ray examination. The cases treated have included several hundred instances of frostbite.

LIEUTENANT-COLONEL JOHN WILLIAM SMITH is honorary surgeon to the Manchester Royal Infirmary, and professor of systematic surgery in the University. An Edinburgh and Manchester man, he qualified M.B., C.M. Edin. with honours in 1886, subsequently becoming an F.R.C.S. Eng. He has had previous military experience, having served with the South African Field Force, and in his early years spent three years as resident surgical officer at the Royal Infirmary, where he is now a distinguished member of the surgical staff. His publications include various studies in anatomy, reports on the condition of the wounded during the Boer War, and an account of six months with a military hospital.

MAJOR FREDERICK HIBBERT WESTMACOTT, who hails from Owens College, Manchester, became an F.R.C.S. Eng. in 1894, and is now honorary assistant aural surgeon and laryngologist to the Royal Infirmary, honorary consulting surgeon to the Manchester Children's Hospital, and consulting surgeon to St. John's Hospital for Diseases of the Ear, Manchester. After qualification Major Westmacott held various resident appointments in Manchester before devoting himself to the special study of the throat, nose, and ear.

CAPTAIN JOHN HEDLEY CROCKER, R.A.M.C.(T.), who has been appointed honorary physician and registrar to the Red Cross Hospital, Richmond, is in private life medical officer of health and school medical officer for Richmond, as well as consulting physician to the Mogden Isolation Hospital. A student of Charing Cross Hospital and Owens College, Manchester, he qualified in 1883, and subsequently became an M.D., Ch.B. Vict., and D.P.H. At one time Captain Crocker was medical officer of health for the Borough of Eccles and Port of Manchester. His extensive experience of public health work naturally renders him a valuable officer in the sanitary service of the Territorial medical department. The institution to which he has been appointed is taking the surplus cases from the 2nd London General Hospital at Chelsea.

VERY satisfactory reports have been made in regard to Queen Mary's Royal Naval Hospital at Southend-on-Sea, which was opened soon after the outbreak of war, and received as its first contingent of sufferers about a hundred Belgian victims. It will be remembered that the hospital has room for nearly three hundred patients, and is under the presidency of Her Majesty the Queen. Miss Finnemore, a "Guy's" sister, who went through the South African War, is the matron in charge, and has been assisted by Mrs. Wells, another "Guy's" sister. The institution has, in addition to an operating theatre, special departments for *x*-ray work and massage.

DR. ROBERT ALEXANDER CHISOLM, M.D., M.R.C.P.Lond., and a Beit Research Fellow, is a member of the staff and resident medical officer to Queen Mary's Naval Hospital. A "Guy's" and Oxford man, he has done valuable research work, particularly in regard to chemical physiology and pathology. Dr. Chisolm is now physician to Queen's Hospital for Children, where he was formerly resident medical officer. At Oxford he won a scholarship at Wadham College, whilst at Guy's Hospital, where he was house physician, he was awarded the Greville Research Studentship. He has also worked at the Evelina Hospital for Sick Children.

THE staff of the Victoria Hospital at Southend-on-Sea form the visiting staff of the Queen Mary's Royal Naval Hospital.

